# Health expenditures spent for prevention, economic performance, and social welfare

**DOI:** 10.1186/s13561-016-0119-1

**Published:** 2016-09-21

**Authors:** Fuhmei Wang, Jung-Der Wang, Yu-Xiu Huang

**Affiliations:** 1 Department of Economics, Department of Public Health, and Research Center for Energy Technology and Strategy, National Cheng Kung University, Tainan, 701 Taiwan; 2 Department of Public Health, College of Medicine, National Cheng Kung University, Tainan, 701 Taiwan; 3 Department of Economics, National Cheng Kung University, Tainan, 701 Taiwan

**Keywords:** Economic growth, Health expenditures spent for prevention, Social welfare

## Abstract

**Background:**

Countries with limited resources in economic downturns often reduce government expenditures, of which spending on preventive healthcare with no apparent immediate health impact might be cut down first. This research aims to find the optimum share of preventive health expenditure to gross domestic product (GDP) and investigate the implications of preventive health services on economic performance and the population’s wellbeing.

**Methods:**

We develop the economic growth model to undertake health-economic analyses and parameterize for Taiwan setting. Based on the US experiences over the period from 1975 to 2013, this research further examines the model’s predictions on the relationship between preventive health expenditure and economic performance.

**Results:**

Theoretical analysis and numerical simulations show that an inverse U-shaped relationship exists between the proportion of GDP spent on prevention and social welfare, as well as between the proportion spent on prevention and economic growth. Empirical analysis shows an under-investment in prevention in Taiwan. The spending of preventive healthcare in Taiwan government was 0.0027 GDP in 2014, while the optimization levels for economic development and social welfare would be 0 · 0119 and 0 · 0203, respectively. There is a statistically significant nonlinear relationship between health expenditure on prevention and the estimated real impact of economic performance from US experiences. The welfare-maximizing proportion of preventive expenditure is usually greater than the proportion maximizing economic growth, indicating a conflict between economic growth and welfare after a marginal share.

**Conclusion:**

Our findings indicate that it is worthwhile increasing investment on prevention up until an optimization level for economic development and social welfare. Such levels could also be estimated in other economies.

## Background

According to the American College of Preventive Medicine (ACPM), the goal of preventive medicine is to protect, promote, and maintain health and well-being and to prevent disease, disability, and death (http://www.acpm.org/page/preventivemedicine). It focuses on the health of individuals, communities, and defined populations. Although they refer these practices to physicians to establish a specialty, it would be generally accepted by health related fields to extend to all those practices by healthcare and public health professionals with the same goal. The Health Division of OECD applies an additional boundary stating that the primary purpose of spending is health, of which we consider as implicit in the definition of the ACPM (https://www.oecd.org/els/health-systems/Expenditure-on-prevention-activities-under-SHA-2011_Supplementary-guidance.pdf). Typical examples of preventive services are early detection of hypertension and diabetes plus reactive prevention and control that would generally reduce complication and/or mortality, which in turn would prolong survival, improve quality of life, and decrease disability under highly cost-effective condition or cost-saving [1, 2]. Although different countries may include expenditures of preventive services with variations (e.g., whether reactive prevention services carried out by primary care physicians are included, etc.), those listed by the National Health Expenditure Accounts in the United States (NHEA) and OECD used for prevention seem to be largely comparable and policy relevant.

Screening and detection of oral, cervical and colorectal cancer at premalignant stage, stage 0, and/or earlier stages would save life-years [3–5], quality of life [6], and healthcare costs [4, 5]. Prevention of end-stage renal disease would save costs of dialysis [7] and long term care [8]. Thus, provision of effective preventive healthcare that reduces the occurrence of catastrophic illnesses would generally reduce expenditures of later diagnosis & treatment for complications and long term care spending for disability. Early health interventions could possibly reap the greatest health benefits in the long run for the population.

Recently, unfavorable macroeconomic environment can have serious immediate as well as long term consequences on mental health and wellbeing across the lifespan and more importantly across generations [9]. The global financial crisis leads many countries to reduce spending dramatically. Countries with limited resources in economic downturns often reduce government expenditures, of which spending on preventive healthcare with no apparent immediate health impact might be cut down first, as there are also sick people in the population requiring immediate treatment. Debate on prevention often focuses on whether the optimal provision of preventive health services exists. Furthermore, most people enjoy health and consumption from other things. “Health and survival are central to the understanding not only of the quality of one’s life, but also for one’s ability to do what one has reason to want to do” [10]. This research provides a framework to incorporate consumption and health with survival to find the optimum share of preventive health expenditure to gross domestic product (GDP) for maximization of population’s wellbeing and economic growth.

Health expenditure in developed countries has risen faster than GDP. The combination of continuously rising healthcare demand and public resource constraints has created a persistent interest in achieving greater efficiency. The preferred measure of health benefits, i.e., the health outcomes themselves, should not be limited to mortality or life expectancy. Instead, we should also consider whether raising the expenditure allocated to preventive healthcare will effectively lead to the attainment of better economic performance and social welfare. Our outcome measures include both life expectancy and utilities of health plus consumption, and thus permit us to examine the lifetime utility after re-allocating medical resources. Based on Taiwan’s experiences, this research calibrates the variations of population’s wellbeing. Moreover, we further corroborate our theoretical implications by empirically examining the U.S. experiences in a longitudinal manner.

## Methods

### The theoretical model

Consider an economy that is populated by households and firms. The representative household lives infinitely and pursues discounted lifetime utility maximization from consumption *C*, and from health, *H*.

1 $$ {\displaystyle \underset{t=\infty }{\overset{t=0}{\int }}\left[ \ln C(t)+\eta \ln H(t)\right]} \exp \left(-\frac{1}{x}t\right)dt\kern1em \upeta >0 $$

in which η presents the positive impact of health on utility. The representative household at a specific age has a life expectancy of *x*, and the reciprocal of this parameter 1/*x*, provides an indication of the mortality rate or the subjective discounting rate [11]. In the following derivation, time scripts have been dropped for simplicity. Individuals demand healthcare services to improve their health and guard against the effects of ill health. The provision of more health services through preventive healthcare *f*, and treatment healthcare *D*, leads to better health [12, 13]:

2 $$ H=\overset{.}{f}+\overset{.}{D} $$

Government could reduce the population’s duration of sick time by devoting treatment medical resources and augment the amount of healthy time by devoting prevention medical resources [14]. The spending on preventive healthcare services could be regarded as the investment on the population’s health, leading to increases in health stock. Diagnostic and therapeutic healthcare expenditure leads to the recovery of health stock and could be regarded as the consumption expenditure as Eq. 2 shows. This research deals with an important policy-issue in health economics, namely the trade-off between prevention and treatment expenditures in health systems. This specification does fit with the general understanding of the relationship between health care, health, and utility. The demand for health care is a derived demand for better health [13]. Households receive positive utilities from both consumption and health. The only reason for demanding healthcare is to improve health.

Barro [15] sets up an endogenous growth model with the flow specification of government expenditure. The public sector provides infrastructures such as highways, airports, and electrical facilities to externally enhance private production. Capital is the only input in production and could be a composite of physical and human capital [16]. The production function is in Cobb-Douglas form and concave with constant returns to scale, presenting that private physical capital (*k*) and public non-health expenditure (*g*) are imperfect substitutes with interior utility-max and growth-max solutions. The household producer possesses the following production technology:

3 $$ y=A{k}^{\alpha }{g}^{1-\alpha } $$

in which *A* is the technological parameter. Parameters α and (1 − α) are the shares of public non-health expenditure and private capital in the goods production and present the degrees to which the public services and private capital affect the productivity. All producers are symmetric, which implies that they set the same price and output in equilibrium. The goods market is one with perfect competition.

To sustain an equilibrium with steady growth, government expenditure could not fix at the exogenous level but should link the scale of an economy [17]. This research is inspired by previous studies [18, 19] and assumes that the government sets its health expenditure on diagnosis & treatment as the fixed fraction of output, *D*_0_, with the probability of ill health (1—*p*) and health expenditure for prevention as the fixed fraction of output, *f*_0_, with the probability of good health, *p* for sustaining an equilibrium in a continual growth framework. The government’s procurement of prevention and treatment adds the stock of prevention and treatment expenditures as the processes in Eqs. 4 and 5 show.

4 $$ \overset{.}{f}={f}_0py $$

5 $$ \overset{.}{D}={D}_0\left(1-p\right)y $$

6 $$ \overset{.}{\underset{t\to \infty }{ \lim }}\left(\frac{\overset{.}{f}}{\overset{.}{D}}\right)=\frac{f_0p}{D_0\left(1-p\right)} $$

Equation 6 assumes that prevention and treatment health services are not reversible [20]. Because prevention and treatment health care are different services, there may request for adjustment costs when prevention (treatment) health services reverse to treatment (prevention) services. Concerning that the probability of becoming healthy affects income [21], the disposable income of the representative household is:

7 $$ {y}_d=p\left(1-\tau -q\right)y+\left(1-p\right)\left(1-\tau -q-\varphi \right)y $$

Households pay income tax at a rateτand health insurance payments at a rate *q* in a NHI (National Health Insurance) system. With a probability of 1-*p*, households fall ill and lose working income, *ϕy*. Disposable income that is not currently consumed becomes capital accumulation:

8 $$ \overset{.}{k}={y}_d-C=\left[1-\tau -q-\varphi \left(1-p\right)\right]y-C $$

The public-sector balanced budget constraint is:

9 $$ g+\overset{.}{f}+\overset{.}{D}+\frac{D_0\left(1-p\right)y}{f_0py}\beta {D}_0\left(1-p\right)y=\left(\tau +q\right)y $$

The government establishes the infrastructure of healthcare system to provide access to healthcare by allocating medical expenditure to satisfy the population’s demands as well as finances its productive expenditure, *g*, an increase in preventive healthcare spending, $$ \overset{.}{f}, $$ an increase in diagnosis & treatment healthcare spending, $$ \overset{.}{D}, $$ and the waste treatment expenditure if early intervention is not implemented through health insurance premium collection, *qy*, and the revenue from income tax, *τy*. Without adequate provision of preventive healthcare, patients with illnesses would generally be diagnosed at a later stage (or even when they have developed further complications), and thus require more expenditure for treatment [22] and may result in a longer duration of disability. The experiences of life years and healthcare expenditures saved from early detection of cervical cancer in Taiwan indicate that prevention interventions provide both health benefits and good use of healthcare resources. The mean lifetime costs of managing stage 0 (US $1316) are significantly lower than those of stages 1–4 of invasive cancer (US $7020, $10,133, $11,120 and $10,015, respectively), indicating the saving of lifetime expenditures for early detection and treatment [4]. Furthermore, the implementation of the 2014 guidelines for hypertension control (namely, reactive prevention) in the U.S. adults between the ages of 35 and 74 years could potentially prevent about 56,000 cardiovascular events and 13,000 deaths annually, while saving future medication costs [1]. With preventive healthcare, the lifetime expenditures of management could be reduced by reactive prevention or avoided altogether because of proactive prevention. The reduction in such costs is captured by the fourth term on the left-hand side of Eq. 9. To present the situation that illnesses are associated with increasing health expenditure, costs of treating illness, *D*_*0*_*y*, are bigger than those of prevention for getting health, *f*_*0*_*y* [23]. A smaller β captures the situation, while a nation is generally healthier, it would usually spend less medical expense for treatment. The wasting cost decreases with the ratio of prevention expenditure and the probability of good health. Providing more and more effective health services for prevention would decrease the risks of diseases and the expenditures for diagnosing and treating them up to a certain marginal effect.

Rearranging government budget constraint, Eq. 9, together with the production function, Eq. 3, generates

10 $$ \frac{g}{k}={\left\{\left(\tau +q\right)-\left[{f}_0p+{D}_0\left(1-p\right)+\frac{D_0\left(1-p\right)}{f_0p}\beta {D}_0\left(1-p\right)\right]\right\}}^{\frac{1}{\alpha }}.{A}^{\frac{1}{\alpha }} $$

The representative household chooses consumption and possesses health stock to maximize the discounted sum of utilities defined in Eq. 1 subject to Eq. 8, and given the initial private capital, *k*_0_. The optimal conditions necessary for this optimization problem are given by Eqs. 11 and 12 together with the household’s budget constraint, Eq. 8:

11 $$ \frac{1}{C}=\frac{\eta }{H\left(1+\theta \right)}=\lambda,\;\theta =\frac{\beta {D}_{{}_0}^2{\left(1-p\right)}^2}{f_0p\left[{f}_0p+{D}_0\left(1-p\right)\right]} $$

12 $$ \frac{\overset{.}{\lambda }}{\lambda }=\frac{1}{x}-\left[1-\tau -q-\phi \left(1-p\right)\right]\eta A{\left(\frac{g}{k}\right)}^{1-\alpha } $$

In these equations, λ is the co-state variable and is the shadow value of the private capital stock measured in utility terms. Differentiating Eq. 11 with respect to time and substituting Eq. 12 into the resulting Eq. 13, determines the growth rate of consumption, which is also the improvement rate of health and the growth rate of income, under our framework.

13 $$ \frac{\overset{.}{C}}{C}=\gamma =\left[1-\tau -q-\phi \left(1-p\right)\right]\alpha {A}^{1/\alpha}\cdot \pi -\frac{1}{x},\;\pi ={\left\{\left(\tau +q\right)-\left[{f}_0p+{D}_0\left(1-p\right)+\frac{D_0\left(1-p\right)}{f_0p}\beta {D}_0\left(1-p\right)\right]\right\}}^{\frac{1-\alpha }{\alpha }} $$

14 $$ \underset{t\to \infty }{ \lim}\lambda (t)k(t)=0 $$

This growth rate depends on the gap between net marginal productivity of capital per capita, the mortality rate, and the time preference rate. Preventive healthcare indirectly affects the growth rate via the ratio of preventive expenditure relative to treatment healthcare expenditure. Equation 14 is the transversality condition, which restricts *k* from growing too fast.

In a standard one-sector endogenous growth model, there is no transitional dynamics, whereby the equilibrium is always on a steady-state balanced growth path [15, 16]. The ratio of government expenditure to physical capital, *g*/*k*, and prevention-output ratio, *f*_0_^*^, are constant. Consumption, *C*, physical capital, *k*, and output, *y*, all grow at a constant rate. In equilibrium, the markets for commodities must be clear. The commodity market is automatically satisfied if we combine the household budget constraint in Eq. (8) and the government budget constraint in Eq. (9), together with Eq. (3). In other words, we have considered the social planner’s objective. Moreover, we are interested in whether the growth-maximization equilibrium of this economy leads to a desirable outcome. Maximizing Eq. 13 with respect to *f*_*0*_ and taking into account the best medical resource allocations yields Eq. 15:

15 $$ \begin{array}{l}\frac{\partial \gamma }{\partial {f}_0}=\left[1-\tau -q-\phi \left(1-p\right)\right]\alpha {A}^{1/\alpha}\pi \hbox{'}\left({f}_0\right)\begin{array}{c}\hfill >\hfill \\ {}\hfill =\hfill \\ {}\hfill <\hfill \end{array}0\kern0.5em \mathrm{if}\;{f}_0^{*}\begin{array}{c}\hfill <\hfill \\ {}\hfill =\hfill \\ {}\hfill >\hfill \end{array}\frac{\sqrt{\beta }D{}_0\left(1-p\right)}{p}\\ {}\pi \hbox{'}\left({f}_0\right)=\frac{1-\alpha }{\alpha }{\left\{\left(\tau +q\right)-\left[{f}_0p+{D}_0\left(1-p\right)+\frac{D_0\left(1-p\right)}{f_0p}\beta {D}_0\left(1-p\right)\right]\right\}}^{\frac{1-2\alpha }{\alpha }}\left[-p+\frac{p\beta {D_0}^2{\left(1-p\right)}^2}{{\left({f}_0p\right)}^2}\right]\end{array} $$

Equation 15 indicates that an increase in the share of preventive healthcare expenditure has an ambiguous impact on the equilibrium growth rate. The key factors for this result are how the diagnosis & treatment healthcare expenditure interacts with the ratio of wasted healthcare expenditure, *β*, the probability of the population with ill health, 1 − *p*, and the probability of the population with good health, *p*. A rise in the share of preventive healthcare expenditure can affect economic performance through two channels. The first is the medical resources crowding out effect, whereby an increase in preventive expenditure reduces expenditure on treatment, as Eq. 9 shows. This channel tends to deteriorate agents’ health status, as Eq. 2 shows, and hence the productivity in an economy. The second is the effects of health improvement, whereby an increase in preventive spending tends to improve agents’ health status. This channel has positive effects on private productivity and leads to better economic performance. The net effect of a rise in preventive expenditure on economic growth depends upon the relative strength of these two channels. Obviously, a rise in the share of preventive expenditure favors (deters) the balanced growth rate if it improves (deteriorates) agents’ health status.

From Eq. 15, we can find a critical value of *f*_*0*_, namely *f*_0_^*^,which maximizes the balanced growth rate:

16 $$ {f}_0^{*}=\frac{\sqrt{\beta }D{}_0\left(1-p\right)}{p} $$

Equation 16 implies that in order to attain economic growth, the optimal share of preventive healthcare relative to output is the fraction of the product of the diagnosis & treatment healthcare share, *D*_*0*_(*1 − p*), and the square root of the share of wasted expenditure to the probability of the population with good health, *p.* When the economy introduces a preventive system, the positive impacts arise through the improved health of the population, and thus its greater productive capacity. Nevertheless, after the critical ratio is reached, the growth effects become negative as the preventive system expands and the treatment healthcare system contracts. The influence of preventive resource use on economic growth is thus non-linear and concave. The health dividend in terms of an enhanced economic growth rate can be achieved only when the initial share of preventive expenditure is smaller than the growth-maximizing share.

We further analyze the influences of medical resource reallocation between prevention and treatment healthcare expenditure on social welfare. This research regards social welfare as the overall welfare of society, specified as the summation of the utilities of all the individuals’ utility functions in the society [24]. Given initial private capital stock *k*_*0*_, both private consumption and health stock grow at a constant rate γ^*^ along the balanced growth path:

17 $$ {C}_t={C}_0 \exp \left({\gamma}^{*}t\right) $$

18 $$ {H}_t={H}_0 \exp \left({\gamma}^{*}t\right) $$

For a given value of *f*_*0*_, Eqs. 16 and 17 have calculated the initial values of consumption and health stock from Eqs. 2 to 8:

19 $$ {C}_0={k}_0\left\{\left(1-\alpha \right)\left[1-\tau -q-\phi \left(1-p\right)\right]{A}^{\frac{1}{\alpha }}{\left\{\left(\tau +q\right)-\left[{f}_0p+{D}_0\left(1-p\right)+\frac{\beta {D_0}^2{\left(1-p\right)}^2}{f_0p}\right]\right\}}^{\frac{1-\alpha }{\alpha }}+\frac{1}{x}\right\} $$

20 $$ {H}_0=\frac{\eta }{1+\theta }{C}_0 $$

Substituting Eqs. 19 and 20 into Eq. 1 yields the household’s welfare function of *f*_*0*_ over an infinite planning horizon:

21 $$ U=x\cdot \left( \ln {C}_0+\eta \ln {H}_0\right)+{x}^2\cdot \left(1+\eta \right)\gamma $$

Substituting the initial values of *C*_*0*_ and *H*_*0*_ in Eqs. 19 and 20 into Eq. 21 and differentiating the resulting equation with respect to *f*_*0*_ yields:

22 $$ \begin{array}{l}\frac{\partial U}{\partial {f}_0}=x\cdot \left[\frac{1}{C_0}\cdot \frac{\partial {C}_0}{\partial {f}_0}+\eta \cdot \frac{1}{H_0}\cdot \frac{H_0}{\partial {f}_0}\right]+{x}^2\left(1+\eta \right)\cdot \frac{\partial \gamma }{\partial {f}_0}\\ {}\kern1.5em =\frac{1}{\left(1+\theta \right)}\left[2{f}^{*}{p}^2+{f}^{*}{D}_0p\left(1-p\right)\right]\beta {D_0}^2{\left(1-p\right)}^2\cdot {H}_0+{x}^2\left(1+\eta \right)\cdot \frac{\partial \gamma }{\partial {f}_0}\end{array} $$

**Fig. 1:**
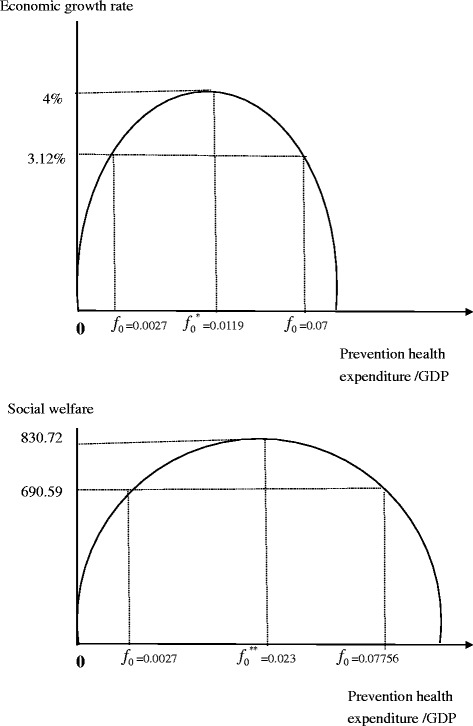
Theoretical and calibrated relationships between the proportion of health expenditures spent for prevention in GDP and economic growth (*upper panel*) versus that and social welfare (*lower panel*)

Equation 22 reveals that the effects of an increase in preventive spending on social welfare include two distinct components. The first term on the right-hand side reflects that a rise in preventive expenditure would be accompanied by an increase in health stock at all points of time, which has a positive effect on social welfare. These effects could be regarded as the *national health effect* related to the investment in prevention programs. The second term on the right-hand side is the *economic growth effect* and presents the ambiguous influences of a rise in prevention health expenditure on the rate of sustained economic growth as Eq. 15 indicates. Equation 22 simultaneously implies that when the government sets its preventive expenditure share at growth-maximizing level f0* (*d*γ*/*df* = 0), a continuing distribution of public spending to the preventive sector at f0** will promote social welfare until maximum. Figure 1(a) and (b) illustrate the conflict between the goals of maximizing economic growth and welfare. Based on Eqs. 16 and 22, the growth-prevention and welfare-prevention nexus could be positive or negative, depending on whether the ratio of prevention spending to GDP galls short of or exceed the critical ratios. An inverse U-shaped relationship exists between the proportion of GDP spent on prevention and social welfare, as well as between the proportion of GDP spent on prevention and economic growth.

### Numerical simulation

This study further provides calibration to present empirical plots of prevention and diagnosis & treatment healthcare provisions versus economic growth and welfare status. The NHI system in Taiwan was launched in 1995, and has become a model that other countries can use when seeking to overhaul their healthcare and medical insurance system. This universal health insurance system covers every citizen. The total health expenditures in Taiwan account for only 7 % of the GDP. Enrollees enjoy almost free access to healthcare, with small co-payments in most clinics and hospitals.

This study has tried to well document and collect the parameter values from peer reviewed publications and real data of Taiwan. The productivity parameter, *A*, has a value of 0.98, which is consistent with the average real per capita economic growth rate of 3.12 % in Taiwan, based on a report from the Council for Economic Planning and Development in 2014. The life expectancy at birth of 80 is from figures produced by the Ministry of Interior in Taiwan in 2014. What discount rate should be used is an old issue in economics [25]. Referring to the concept used in epidemiology [26, 27], the longer the life expectancy, the smaller the mortality rate, and the lower the discount rate, and vice versa. The degree of public services, 1-α, affecting external technology is set at 0.5 [28]. The income tax rate, NHI premium, the income loss rate with illness, and the wasted treatment expenditure ratio are collected from the Ministry of Finance and the Ministry of Health and Welfare in Taiwan in 2014, and are set at 13.35 %, 4.91 %, 50 %, and 0.65, respectively.

The impact of health on utility is set at 0.48 [29]. The shares of preventive and treatment healthcare expenditure in relation to GDP are collected from the Ministry of Health and Welfare in Taiwan in 2014 and set at 0.27 and 6.3 %, respectively. Table 1 presents the parameter settings. Based on the benchmark parameter values, Eqs. 13, 19, 20 and 21, and given the initial capital stock of 1000, the equilibrium economic growth rate, the growth-maximization preventive expenditure share, the welfare-maximizing preventive expenditure share, the initial values of consumption and health stock, and welfare are calibrated as γ* = 3.12 %, *f*_0_^*^ = 0.0119, *f*_0_^**^ = 0.0203, C_0_ = 57.94, H_0_ = 6.73, and U = 690.59, respectively. According to Fig. 1, economic growth and welfare will be maximized at 4 % and 830.72 when the preventive expenditure is allocated at the optimum shares, instead of the actual economic growth and welfare, which are at 3.12 % and 690.59.

**Table 1:** Parameter Values for the Calibrated Economy

Production parameters	*A* = 0.98; *α* = 0.5; *k*_*0*_ = 1000
Utility parameters	*η* = 0.48; *x* = 80
Healthcare parameters	*f*_*0*_ = 0.0027; *D*_*0*_ = 0.063
Government sector	∅ = 0.5;*q* = 0.0491 *τ* = 0.1335; *β* = 0.65

**Fig. 2:**
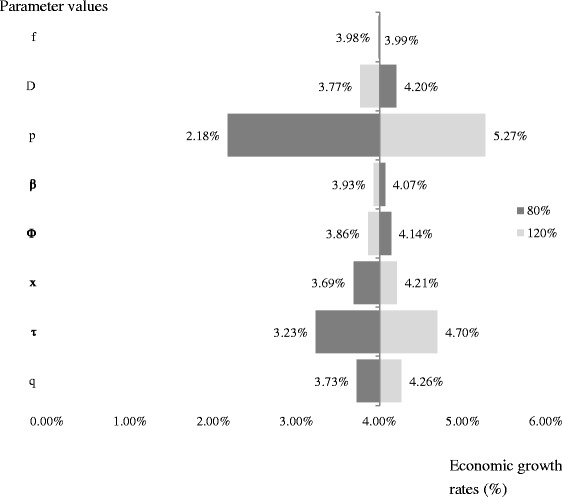
Sensitivity analysis of varying parameter values on economic growth

**Fig. 3:**
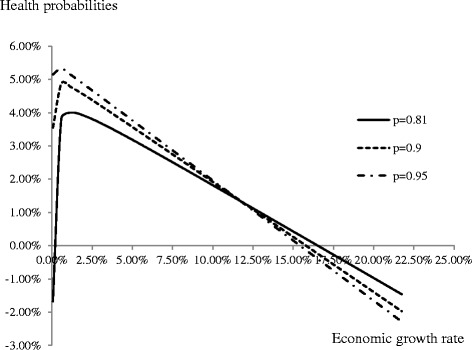
Calibrated relationship at different health probabilities between the proportion of health expenditures spent for prevention in GDP and economic growth

Based on the methods of the sensitivity analysis [30], when the value of one specific parameter is increased or decreased by 20 %, the values of other parameters are kept at their benchmark values. The changes in the health probabilities are the main drivers of the changes in the economic growth rate as Fig. 2 presents. The growth behavior of the system depends on the value of the probability of the population being in good health as Fig. 3 shows. However, the trends do not change. Parameter changes result in the variations of social welfare but do not affect the theoretical findings. Therefore, the related figures are not provided here.

Numerical simulations report that the optimal preventive expenditure shares for maximizing economic growth and social welfare would be 0.0119 and 0.0203, respectively, instead of the 0.0027 that is actually allocated as Fig. 1(a) and (b) show. The welfare-maximizing preventive expenditure share would be greater than the growth-maximizing one, and so there is a conflict between economic growth and welfare after the optimal investment of 0.0119 GDP. Taiwan’s actual preventive healthcare services are thus underprovided.

Now consider a government that has some targets of economic growth rates and social welfare that it needs to achieve with prevention services. Assume that the economic growth rate and social welfare are less than the maximum feasible rates of prevention. Then, as Fig. 1(a) and (b) show, there are two rates of prevention/GDP that can achieve the current economic growth rates and social welfare. With real one, 0.0027, prevention services are underfunded and the economic growth rate is 3.2 % as well as social welfare is 690.59; with the other, preventive services are overfunded at 0.07 and 0.07756 with the same economic growth rate and social welfare as those of underfunded situations. Preventive healthcare services have crowded out treatment services provision and hurt the population’s health, in turn reduced the economy’s productivities and deteriorated social welfare. We do not appear to observe such conditions in practice.

### Empirical application: estimating the quantitative importance of prevention health expenditure on economic performance in the U.S

We search PubMed, Medicine, EconLit, and Google Scholar for empirical studies published between November 15 2009, and November 15 2015, with search terms of “prevention and economic growth”, “prevention and social welfare”, and “prevention and macroeconomics”. We found many prevention studies have attempted to present the healthcare expenditures saved from early detection of specific diseases [1, 4, 5]. There are also many studies conducted evidence-based analyses for efficient healthcare — whether prevention or treatment — and then encourage the appropriate delivery of efficient interventions [2, 31]. In addition to determining which preventive interventions are most efficient, there is a need to optimally allocate health expenditures that are not yet fully deployed and that could serve the whole population and bring about substantial aggregate improvements in health and wellbeing in a growing economy.

Although this research made use of actual parameter values related to the domestic economy, the theoretical model has been built in a manner that practically can be used to depict any type of economy. Therefore, as a robustness check, we would like to test whether the results receive further empirical support based on parameters that have been explicitly measured through estimations longitudinally. This not only will corroborate the original findings, but also will provide sound empirical support to the validity of the theoretical model. We have not chosen Taiwan’s data for such a test because the official statistics of public health were published over the period from 2000 to date. With few time periods estimators, ordinary least square (OLS) estimates would be biased.

Based on the U.S. experiences over the period from 1975 to 2013, this research examines the model’s predictions on the relationship between preventive health expenditure and economic performance. The data are collected from NHEA, the official estimates of total health care spending in the United States. The prevention health expenditures are composed of national health expenditures on public health, worksite healthcare, maternal and child health, substance abuse and mental health services administration (SMHSA), and school health.

### Research design

1. Given the finite resources available for healthcare spending, this research aims to examine the influences of raising preventive health expenditure on economic performance and social welfare.
2. We integrate the satisfaction concept of utility in economics with life expectancy, to highlight the effectiveness of prevention in macroeconomic terms.
3. Empirical analysis shows that the investment of prevention in Taiwan government was 0.0027 GDP in 2014, while the optimization levels for economic development and social welfare would be 0 · 0119 and 0 · 0203, respectively.
4. Estimation results of US experience over the period from 1975 to 2013 corroborate our theoretical implication of optimally allocating health expenditures on prevention.

## Results

**Fig. 4:**
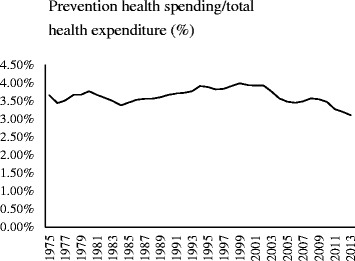
The share of preventive health spending to total health expenditure in the U.S. over the period from 1975 to 2013

The above theoretical derivation indicates that it is worthwhile increasing investment on prevention up until an optimization level for economic development and social welfare, as empirically calibrated based on Taiwan Economy (Eq. 22 and Figs. 1, 2 and 3). Figure 4 presents the historical trend of U.S. prevention health expenditures (inflation-adjusted). Prevention’s share of total health expenditures rose from 3.65 % in 1975 to 3.99 % in 1999, then fell to 3.09 % in 2013. Prevention health expenditure have declined and could undermine prevention and weaken responses to health inequalities and new health treats [32].

Estimation results through model construction show a strongly non-linear relationship between the ratios of preventive health expenditure to GDP and log GDP per person. The corresponding regression (with a quadratic term included to account for the nonlinearity) is

23 $$ y=\underset{(0.062)}{8.631}+\underset{(37.191)}{555.026}{f}_0-\underset{(4798.075)}{34042.04}{f}_0^2\kern1em {R}^2=0.98 $$

where y is log GDP per person and the numbers in parentheses are standard errors. Provide 99 % confidence intervals for estimates. The estimates imply that ∂*y*/∂*f*_0_ = 555.026 − 2 × (34042.04)*f*_0_, which is positive for ${f}_0<555.026/\left[2\left((34042.04)\right]\right.\cong 0.815\%={f}_0^{*}$ and negative for ${f}_0>{f}_0^{*}$ Thus, there is a statistically significant nonlinearity relationship between health expenditure on prevention and the estimated real impact of economic performance. Currently, actual ratio of prevention health expenditure to GDP is 0.384 %. Accordingly, the estimated current unmet demand for prevention devotion is more than two times that currently being provided. Allocating more resources to effective prevention programs would lead to the attainment of better economic performance and welfare position, which would be accompanied with better health for the population. We find that the US experiences corroborate our theoretical implication of optimally allocating health expenditures on prevention.

## Conclusions

Existing studies focused mostly on analyzing the costs and effectiveness/benefits of specific interventions or treatments. This research provides a more paternalistic guidance of medical resource allocations. With appropriate healthcare expenditure allocation, the population could have better health and stronger human capital to contribute to economic growth through improved productivity plus reduced future demands on healthcare. Effective health interventions are possibly associated with faster economic growth and higher welfare status. Recent economic downturns tend to have the greatest effects on working age adults. The World Health Organization has raised concern over the crisis’ impact on global health to closely monitor and protect health, in particular among the poor and vulnerable people [33]. Based on the main function of health expenditure in OECD Health Statistics, public health expenditures are composed of expenditures on inpatient care, outpatient care, long-term care, pharmaceuticals, prevention/public health, and administration. Global economic downturn leads to slow growth of expenditures on inpatient care, outpatient care, long-term care, and even negative growth of prevention/ public health expenditure over the period of 2008–2011 [9, 34, 35].

The overarching research question which is posed by this research is a pertinent one, especially in the context of currently financially constrained health systems around the world, which are being squeezed by multiple developments including (1) a financial squeeze (due to the global economic downturn), (2) rapid growth of expensive treatment technology such as molecular target therapy for cancer, chemotherapy of hepatitis C, etc. and (3) quickly ageing populations. Hence, it is crucial (more than ever) to pursue proper policy analysis at the macroeconomic level, which will allow policymakers to properly allocate scarce resource across public healthcare sectors, including prevention and treatment. Our framework is expected to empirically applicable to economies with and without universal coverage of health services.

Over the coming several decades, both developing and industrialized countries will face sharp rises in health expenditure, as well as other long-term care challenges because of the aging population [32]. Given the finite resources available for healthcare spending, we should ask whether preventive health services are having the desired impact on a nation’s health and economic performance. Theoretical analysis and numerical simulations using data from Taiwan suggest that, raising the expenditure allocated to effective prevention programs would lead to the attainment of better health for the population, faster economic growth and higher social welfare. Namely, the provision of preventive healthcare interacts positively with a socioeconomic perspective, and it is worthwhile increasing investment on prevention up until an optimization level for economic development and social welfare, respectively (Eq. 22), which is first verified in an economy with universal health services, i.e. Taiwan (Figs. 1, 2 and 3), and further corroborated longitudinally based on the US experiences (Fig. 4 and Eq. 23). Studies also show every US dollar invested in proven, community-based public health efforts saves 5.60 US dollars in future health care costs [36]. Thus, we conjecture that the same implications could be applicable to other countries that have or are developing a system of universal coverage to all the people resided in each country.
